# Rituximab-Induced Coronary Vasospasm

**DOI:** 10.1155/2012/984986

**Published:** 2012-06-06

**Authors:** Linda Lee, Vishal Kukreti

**Affiliations:** ^1^Department of Oncology, Niagara Health System, St. Catharines, ON, Canada L2R 2Z7; ^2^Department of Medical Oncology & Hematology, Princess Margaret Hospital, Room 5-227, 610 University Avenue, Toronto, ON, Canada M5G 2M9

## Abstract

Rituximab has improved the treatment of B-cell non-Hodgkin lymphomas. While it is generally well tolerated, serious adverse effects including infusion reactions with hemodynamic compromise and cardiac arrhythmias or ischemia are possible. We report a case of coronary vasospasm occurring during a rituximab infusion in a patient with minimal tumour burden and who had no cardiac risk factors. This case highlights that determination of the cause of ischemia is important and may identify some patients who can be successfully rechallenged.

## 1. Introduction

Rituximab is a chimeric murine-human monoclonal antibody that binds specifically with CD20, which is expressed on the majority of mature B-cell non-Hodgkin lymphomas. In the last decade, use of this drug alone or in combination with chemotherapy has improved the outcomes of patients with these diseases. The most common adverse effect is immediate hypersensitivity reactions, but postmarketing reports have cited increased rates of rare but serious complications such as hepatitis reactivation, bowel perforation or obstruction, progressive multifocal encephalopathy, and cardiac arrhythmias or ischemia [1].

 We report on the case of documented coronary vasospasm during a rituximab infusion, occurring in the absence of other symptoms typical of an infusion reaction and in a patient with no coronary artery disease.

## 2. Case Presentation

 A 52-year-old woman presented with epigastric pain, melena, and a five-kilogram weight loss. She has a past history of gastric ulcers and her father had died of gastric cancer. She had no anemia, but symptoms were persistent despite therapy with a proton pump inhibitor so she underwent a gastroscopy which revealed only a healing ulcer in the duodenum. Biopsy revealed follicular lymphoma (FL), grade 1-2. There was no dysplasia or *Helicobacter pylori* infection.

 Staging computed tomography (CT) revealed only thickening of the second part of the duodenum with no significantly enlarged lymph nodes. Bone marrow biopsy was positive for involvement with FL. For symptomatic stage 4 disease, systemic treatment with rituximab, cyclophosphamide, vincristine, and prednisone (R-CVP) was recommended. Screening blood work revealed a history of hepatitis B infection with a positive core antibody so she was started on lamivudine for reactivation prophylaxis.

**Figure 1:**
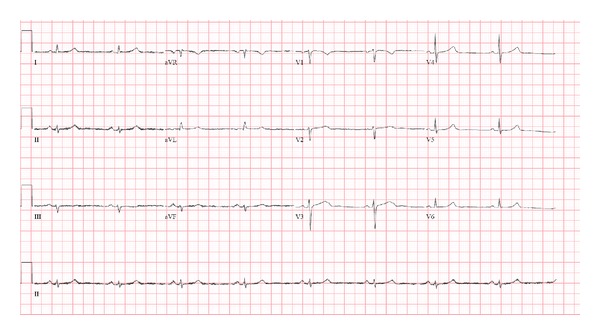
Baseline ECG obtained at initial consultation visit.

**Figure 2:**
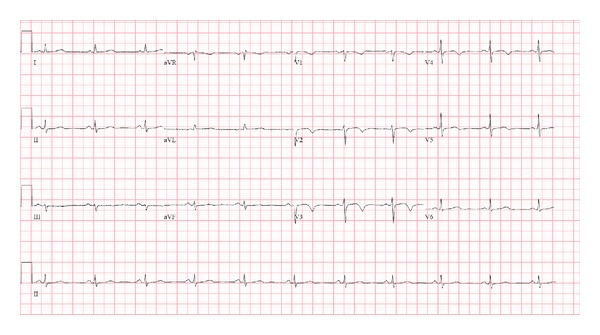
ECG obtained while patient experienced chest pain during initial rituximab infusion, which demonstrates new-onset deep T-inversion in anterior precordial leads V2 to V5.

Following administration of standard premedication with diphenhydramine 25 mg iv, acetaminophen 1000 mg po, and prednisone 100 mg po, the rituximab infusion was initiated prior to the other antineoplastic agents. Within 10 minutes of her first treatment, she developed retrosternal chest pain associated with dyspnea. She had no hypotension, fever, rigors, nausea, or skin changes. Vital signs were stable and unchanged from baseline. The infusion was held, nitroglycerin was given, and pain resolved after 15 minutes. She has no cardiac risk factors other than a seven pack-year smoking history, but ECG documented new onset T-wave inversion in the anterior precordial leads (Figures 1 and 2). She was admitted to hospital and started onto aspirin, clopidogrel, and low-molecular-weight heparin. Subsequent cardiac enzymes were negative. Cardiac catheterization revealed normal cardiac function with no evidence of occlusive disease in the coronary arteries.

Consequently, she was rechallenged with rituximab with continuous cardiac monitoring. Intravenous dexamethasone 8 mg was given along with standard premedication with diphenhydramine, acetaminophen, and prednisone. There was no recurrent chest pain or ECG changes. Restaging CT showed a complete response, and she remains on maintenance rituximab.

## 3. Discussion

Four clinical trials have demonstrated that the addition of rituximab to chemotherapy results in improved survival in the first-line treatment of FL [2–5]. While it is generally well tolerated, rituximab has been associated with infusion reactions including fever, chills, hypotension, and dyspnea. Incidence of infusion-related side effects is highest following the first dose of rituximab and is related to underlying tumour burden [6]. Severity of infusion reactions is correlated with release of cytokines (namely, TNF-*α*, IL-6, and IL-8) and activation of the complement system [7, 8]. Pre-treatment using acetaminophen, antihistamines, and steroids reduces the incidence of these reactions.

Cardiac events including hypotension, arrhythmias, and chest pain were observed in at least two previous phase II trials of rituximab alone but were attributed either to previously existing heart disease [9] or infusion reactions [2]. To examine the latter, a phase I-II study examining infusion rates and cardiac function revealed only one case of asymptomatic changes on electrocardiogram [10], but this study also excluded patients with any preexisting cardiac conditions. Acute coronary ischemia precipitated by rituximab in those with a history of cardiac disease or those with cardiac risk factors has been described in recent case series and case reports [11, 12].

However, our study is the first to demonstrate an acute coronary syndrome (ACS) due to coronary vasospasm. Although chest pain resolved following discontinuation of the infusion other features typical of a hypersensitivity reaction were absent. Symptoms were responsive to nitroglycerin and were associated with reversible ECG changes and no cardiac enzyme elevation. She had minimal tumour burden, no significant cardiac risk factors, and normal coronary anatomy with no atherosclerotic disease.

Coronary vasospasm has not been described during rituximab infusion but is a well-known side effect of continuous 5-fluorouracil infusion, which presents in a similar manner [13–15]. The mechanism for 5-fluorouracil cardiotoxicity is not known, but it is possible to re-challenge patients with reduced doses and use of prophylactic nitroglycerin [16]. In our case, the vasospasm occurring within minutes of the infusion was likely due to the same mechanism driving other infusion-related reactions, so intravenous corticosteroids and antihistamines were given prior to successful rechallenge.

## 4. Conclusion

This case highlights coronary vasospasm as a previously unreported infusion-related adverse effect of rituximab. In patients who develop chest pain during rituximab administration, this possibility of ACS should be considered and appropriate cardiac investigation should be undertaken, even if patients have no history of or risk factors for cardiac disease. In the absence of significant coronary artery disease, rechallenging with rituximab may be possible following optimal premedication in a monitored setting in selected cases where benefit of rituximab overweighs the risk of recurrent of cardiac ischemia.
